# O Sexo Feminino está Associado à Mortalidade na Cirurgia de Revascularização do Miocárdio?

**DOI:** 10.36660/abc.20240664

**Published:** 2025-05-08

**Authors:** Maxim Goncharov, Erlon Oliveira de Abreu Silva, Pedro Gabriel Melo de Barros e Silva, Fabiane Letícia de Freitas, Adriana Costa Moreira, Lucas Tramujas, Alexandre Biasi Cavalcanti, Ieda Maria Liguori, Fabio Biscegli Jatene, Ieda Biscegli Jatene, Claudia Maria Rodrigues Alves

**Affiliations:** 1 Hospital do Coração São Paulo SP Brasil Hospital do Coração, São Paulo, SP – Brasil; 2 Hospital das Clínicas Faculdade de Medicina Universidade de São Paulo São Paulo SP Brasil Instituto do Coração do Hospital das Clínicas da Faculdade de Medicina da Universidade de São Paulo, São Paulo, SP – Brasil

**Keywords:** Mortalidade Hospitalar, Procedimentos Cirúrgicos Cardiovasculares, Mulheres

## Abstract

**Fundamento:**

Mulheres submetidas à cirurgia de revascularização do miocárdio (CRM) apresentam piores desfechos intra-hospitalares, mas não está claro se essas diferenças são atribuídas ao sexo ou a fatores clínicos.

**Objetivo:**

Comparar os desfechos intra-hospitalares entre mulheres e homens submetidos à CRM.

**Métodos:**

Este estudo observacional, unicêntrico e retrospectivo analisou dados de 9.845 pacientes submetidos à CRM entre 1995 e 2022; dentre eles, 1.947 (19,8%) eram mulheres. Para avaliar o sexo feminino como fator prognóstico de mortalidade intra-hospitalar, foram realizadas análises estatísticas descritivas, regressões logísticas univariada e multivariada e pareamento por escore de propensão. O nível de significância foi estabelecido em 5%.

**Resultados:**

Mulheres apresentaram maior idade (66,7 vs 62,19 anos, p<0,001), menor índice de massa corporal (26,91 vs 27,64, p<0,001) e maior prevalência de diabetes melito (34,0% vs 31,6%, p=0,045). Elas também tiveram maior tempo de internação (14,84 vs 13,13 dias, p<0,001) e maior mortalidade operatória (4,8% vs 2,4%, p<0,001). Após regressão logística, o sexo feminino foi associado à maior mortalidade (OR: 1,51, IC 95%: 1,15-1,99, p=0,003). Após o pareamento, não houve diferença significativa na mortalidade (OR: 1,20, IC 95%: 0,88-1,64, p=0,241), porém o tempo de internação permaneceu maior entre as mulheres.

**Conclusão:**

Ao se igualarem os fatores clínicos entre homens e mulheres por meio do pareamento, as diferenças na mortalidade desaparecem. Isso sugere que intervenções voltadas à redução das disparidades podem ser eficazes para melhorar os desfechos de mortalidade em mulheres submetidas à CRM.

## Graphical abstract

**Figura Central:**
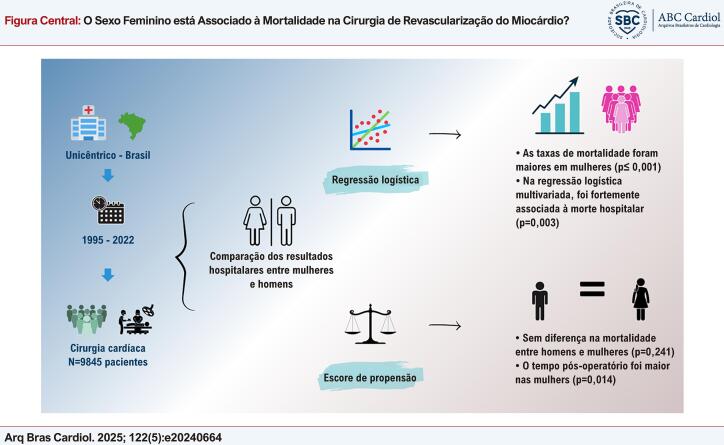
O Sexo Feminino está Associado à Mortalidade na Cirurgia de Revascularização do Miocárdio?

## Introdução

A doença cardiovascular é a principal causa de morbimortalidade no mundo, sendo a doença arterial coronariana (DAC) sua manifestação mais comum.^1^ Em países em desenvolvimento, a interpretação dos resultados de registros nacionais é desafiadora devido à heterogeneidade das populações e às diferenças significativas entre hospitais públicos e privados.^2-4^

Ainda são escassos os dados disponíveis sobre esse tema na população brasileira. Atualmente, 58% dos procedimentos de revascularização são realizados por angioplastia, e a proporção entre angioplastia e cirurgia de revascularização aumentou significativamente entre 2008 e 2019. Essa mudança reflete um novo perfil de pacientes, com características menos favoráveis à cirurgia, o que pode impactar os resultados da cirurgia de revascularização do miocárdio (CRM).^5-8^

As doenças do aparelho circulatório foram responsáveis por mais de 170.000 mortes de mulheres em 2019.^1^ O sexo tem se mostrado um fator importante no tratamento da cardiopatia isquêmica. De modo geral, as mulheres tendem a desenvolver a doença mais tardiamente devido à proteção hormonal, o que contribui para uma maior mortalidade na CRM.^9-13^ No entanto, alguns estudos não identificaram essa diferença.^14^ Essa discrepância pode ser explicada pela exclusão de fatores específicos ao sexo — como menopausa precoce, disfunção microvascular e diferenças no diâmetro arterial — em grandes registros e estudos randomizados.^15-17^

Dada a alta incidência de DAC, especialmente entre mulheres, é essencial gerar mais dados nacionais para preencher a lacuna de conhecimento sobre essas diferenças. Assim, o presente estudo teve como objetivo comparar as características e os desfechos demográficos, clínicos e cirúrgicos entre homens e mulheres, além de avaliar o impacto do sexo feminino como fator prognóstico independente para a mortalidade na CRM.

## Métodos

### Desenho do estudo

Trata-se de um estudo observacional e retrospectivo, no qual foi analisada uma coorte de pacientes submetidos à CRM em um centro cardíaco privado (Figura Central).

Desde 1995, os dados de todos os procedimentos cirúrgicos realizados em pacientes com mais de 18 anos submetidos à CRM — eletiva ou não, isolada ou associada a procedimento valvar, aneurisma ventricular ou cirurgia da aorta ascendente — são coletados diariamente por profissionais de saúde. Os dados foram armazenados em uma plataforma eletrônica integrada ao sistema Tasy^®^ de prontuário hospitalar (Philips, Blumenau, SC, Brasil). Os dados foram armazenados no servidor do hospital, com acesso restrito aos profissionais da própria instituição.

**Figura 1:**
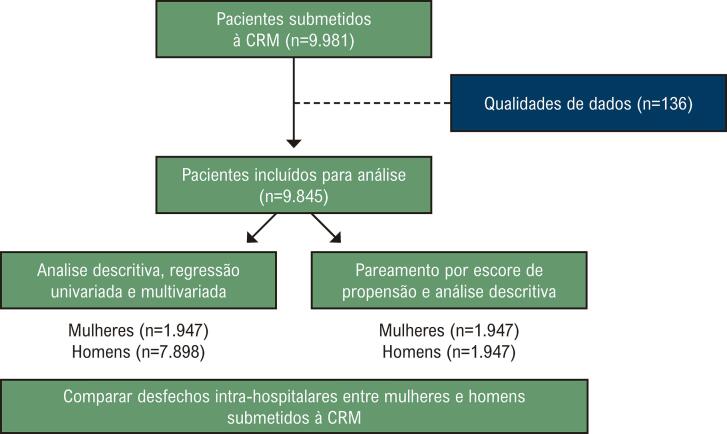
– Fluxograma de seleção de pacientes para inclusão no estudo. CRM: cirurgia de revascularização do miocárdio.

Para garantir a fidelidade e a qualidade dos dados, foi realizada uma auditoria indireta por meio da avaliação de consistência, completude e acurácia das informações. Além disso, a incidência de características perioperatórias foi analisada ano a ano para assegurar a credibilidade das variáveis. Como resultado, 136 pacientes com dados incompletos foram excluídos da análise final (Figura 1).

### Análise estatística

Todas as análises foram realizadas utilizando o software R, versão 4.0.1.

As variáveis categóricas foram descritas em frequências absolutas e percentuais, enquanto as variáveis contínuas foram apresentadas como média e desvio padrão (DP). A normalidade das variáveis contínuas foi avaliada por meio do teste de Anderson-Darling; a homogeneidade das variâncias, pelo teste de Levene.

Na comparação entre os grupos, todas as variáveis contínuas apresentaram distribuição normal e foram avaliadas por meio do teste t de Student não pareado para comparação das médias. As variáveis categóricas foram comparadas utilizando o teste exato de Fisher ou o teste do qui-quadrado, conforme apropriado.

Uma análise de sensibilidade adicional foi realizada com o uso do pareamento por escore de propensão, com o objetivo de minimizar possíveis vieses entre os grupos analisados. As variáveis incluídas no pareamento foram idade, índice de massa corporal (IMC), cirurgia associada, doença pulmonar obstrutiva crônica (DPOC), urgência ou emergência, infarto do miocárdio (IM) nos últimos 30 dias, classe de angina segundo classificação da *Canadian Cardiovascular Society* (CCS), classe de dispneia segundo classificação da *New York Heart Association* (NYHA), doença arterial periférica, CRM prévia, insuficiência renal crônica e classe de fração de ejeção.

Por fim, regressões logísticas univariada e multivariada foram conduzidas para identificar fatores de risco independentes para mortalidade intra-hospitalar, considerando o sexo feminino como uma das variáveis de risco, juntamente com os demais fatores. O nível de significância foi estabelecido em 5%.

## Resultados

Foram observadas diferenças significativas nas características basais entre homens e mulheres (Tabela 1). Mulheres apresentaram maior idade, menor IMC e maior prevalência de hipertensão arterial sistêmica (HAS) e diabetes melito (DM). Além disso, exibiam classes funcionais mais avançadas de dispneia e angina, porém menor ocorrência de IM recente, insuficiência renal crônica e cirurgias cardíacas prévias, em comparação aos homens.

**Tabela 1:** – Características pré-operatórias dos pacientes submetidos à cirurgia de revascularização do miocárdio

Características	Mulheres (n=1.947)		Homens (n=7.898)		Valor p
Idade (anos), média ± DP	66,7±10,03		62,19±10,15		<0,001
Altura (m), média ± DP	1,57±0,07		1,71±0,07		<0,001
Peso (kg), média ± DP	66,76±12,3		80,85±13,81		<0,001
IMC, média ± DP	26,91±4,87		27,64±4,28		<0,001
Uso de tabaco, n (%)	322	(16,5)	2.133	(27)	<0,001
HAS, n (%)	1.560	(80,1)	5.662	(71,7)	<0,001
DM, n (%)	662	(34,0)	2.499	(31,6)	0,045
Dislipidemia, n (%)	1.166	(59,9)	4.603	(58,3)	0,200
DPOC, n (%)	18	(0,9)	110	(1,4)	0,130
Classe CCS de angina, n (%)					
Classe 1	745	(38,3)	3.635	(46,0)	<0,001
Classe 2	507	(26,0)	1.993	(25,2)	
Classe 3	295	(15,2	1.099	(13,9)	
Classe 4	400	(20,5)	1.171	(14,8)	
Classe NYHA de dispneia, n (%)					
Classe I	972	(49,9)	4.565	(57,8)	<0,001
Classe II	579	(29,7)	2.124	(26,9)	
Classe III	314	(16,1)	1.028	(13,0)	
Classe IV	82	(4,2)	181	(2,3)	
IM prévio (30 dias), n (%)	550	(28,2)	2.538	(32,1)	0,001
Insuficiência renal crônica (creatinina >2 mg/dL)	49	(2,5)	274	(3,5)	0,040
Doença arterial periférica, n (%)	19	(1,0)	77	(1,0)	0,900
AVC prévio, n (%)	65	(3,3)	211	(2,7)	0,100
CRM prévia, n (%)	150	(7,7)	787	(10,0)	0,002
Uma reoperação, n (%)	138	(7,1)	710	(9,0)	0,020
Duas ou três reoperações, n (%)	12	(0,6)	76	(1,0)	
Classe de fração de ejeção, n (%)					
Normal (>55%)	1.200	(61,6)	4.778	(60,5)	0,550
Leve (54-45%)	374	(19,2)	1.506	(19,1)	
Moderada (44-35%)	278	(14,3)	1.231	(15,6)	
Grave (<35%)	95	(4,9)	382	(4,8)	

AVC: acidente vascular cerebral; CCS: Canadian Cardiovascular Society; CRM: cirurgia de revascularização do miocárdio; DM: diabetes melito; DPOC: doença pulmonar obstrutiva crônica; DP: desvio padrão; HAS: hipertensão arterial sistêmica; IM: infarto do miocárdio; IMC: índice de massa corporal; NYHA: New York Heart Association.

As características intraoperatórias (Tabela 2) revelaram maior frequência de cirurgias não eletivas e de procedimentos associados entre as mulheres, especialmente os valvares. As mulheres também apresentaram menor uso de circulação extracorpórea. Complicações intraoperatórias como arritmias, sangramento e síndrome de baixo débito cardíaco foram mais comuns no grupo feminino.

**Tabela 2:** – Características intraoperatórias dos pacientes submetidos à cirurgia de revascularização do miocárdio

Características intraoperatórias	Mulheres (n=1.947)		Homens (n=7.898)		Valor p
	n	%	n	%	
Status de urgência ou emergência	81	4,2	220	2,8	0,002
Cirurgia associada	337	17,3	908	11,4	<0,001
Válvula	209	10,7	534	6,8	<0,001
Aorta	2	0,1	1	0,01	0,100
Aorta e válvulas	0	0	2	0	1
Outras cirurgias	126	6,5	371	4,7	<0,001
Utilização de circulação extracorpórea	1.825	93,7	7.504	95	0,020
Complicações intraoperatórias	222	11,4	764	9,7	0,020

As complicações pós-operatórias (Tabela 3) foram semelhantes entre homens e mulheres quanto ao tempo de permanência na UTI. No entanto, as mulheres apresentaram um tempo de internação hospitalar mais prolongado. Embora a proporção geral de complicações pós-operatórias tenha sido comparável entre os grupos, a mortalidade intra-hospitalar foi significativamente maior entre as mulheres.

**Tabela 3:** – Complicações pós-operatórias dos pacientes submetidos à cirurgia de revascularização do miocárdio

Complicações pós-operatórias	Mulheres (n=1.947)		Homens (n=7.898)		Valor p
Complicações totais, n (%)	735	(37,8)	2.804	(35,5)	0,060
Embolia venosa, n (%)	21	(1,1)	77	(1,0)	0,670
Balão intra-aórtico, n (%)	56	(2,9)	154	(1,9)	0,010
Coagulopatia, n (%)	18	(0,9)	74	(0,9)	0,900
IM durante a internação, n (%)	80	(4,1)	268	(3,4)	0,130
Coma, n (%)	16	(0,8)	10	(0,1)	<0,001
AVC, n (%)	105	(5,4)	230	(2,9)	<0,001
Insuficiência cardiorrespiratória, n (%)	29	(1,5)	74	(0,9)	0,030
Duração da permanência hospitalar (dias), média ± DP	14,84±10,96		13,13±8,5		<0,001
Dias após a cirurgia, média ± DP	10,84±9,53		9,73±7,19		<0,001
Duração da permanência na UTI (horas), média ± DP	85,4±133,42		74,85±84,61		0,400
Óbito, n (%)	93	(4,8)	192	(2,4)	<0,001

AVC: acidente vascular cerebral; DP: desvio padrão; IM: infarto do miocárdio; UTI: unidade de terapia intensiva.

A regressão logística univariada revelou que variáveis como sexo, idade, IMC, cirurgia associada, status de urgência ou emergência, IM recente, classe de angina, dispneia, CRM prévia, DPOC, insuficiência renal crônica e classe de fração de ejeção foram estatisticamente significativas (Tabela Suplementar 2). A doença arterial periférica não teve significância estatística, mas foi incluída na análise multivariada juntamente com as demais variáveis significativas. Com base nesses achados e nos principais modelos de risco (EuroSCORE II, SinoSCORE e InsCor) (Tabela Suplementar 3), 13 variáveis foram selecionadas para a regressão logística multivariada (Tabela 4).

**Tabela 4:** – Regressão multivariada em pacientes submetidos à cirurgia de revascularização do miocárdio

Variável	OR	IC 95% (limite inferior)	IC 95% (limite superior)	Valor p
Idade (anos)	1,05	1,04	1,07	<0,001
Sexo (feminino)	1,52	1,15	1,99	0,003
IMC	0,96	0,94	0,99	0,007
Cirurgia associada	3,02	2,28	3,98	<0,001
DPOC	2,47	1,11	4,86	0,015
Status de urgência ou emergência	3,7	2,34	5,69	<0,001
IM recente (30 dias)	1,15	0,87	1,51	0,322
Classe CCS de angina, n (%)				
Classe 2	1,11	0,78	1,56	0,568
Classe 3	1,27	0,87	1,84	0,204
Classe 4	1,6	1,14	2,23	0,006
Classe NYHA de dispneia, n (%)				
Classe II	1,12	0,81	1,54	0,494
Classe III	1,31	0,91	1,87	0,136
Classe IV	2,48	1,55	3,9	<0,001
Doença arterial periférica	1,92	0,71	4,33	0,153
CRM prévia	2,14	1,56	2,89	<0,001
Classe de fração de ejeção				
45-54%	1,24	0,86	1,76	0,24
35-44%	1,74	1,24	2,43	0,001
<35%	3,4	2,26	5,08	<0,001
Insuficiência renal crônica (creatinina >2 mg/dL)	1,91	1,17	2,99	0,007

CCS: Canadian Cardiovascular Society; CRM: cirurgia de revascularização do miocárdio; DPOC: doença pulmonar obstrutiva crônica; IM: infarto do miocárdio; IMC: índice de massa corporal; NYHA: New York Heart Association; OR: odds ratio.

Após a regressão logística multivariada, variáveis como idade, cirurgia associada, status de urgência ou emergência, IM recente, classe de angina, dispneia, CRM prévia, DPOC, insuficiência renal crônica e classe de fração de ejeção foram associadas a um maior risco de mortalidade. O IMC foi um fator protetor, enquanto o sexo feminino também foi significativamente associado a maior mortalidade.

Após o pareamento por escore de propensão, com 1.947 pacientes em cada grupo, a maioria das características pré-operatórias e intraoperatórias foi semelhante, exceto o uso de tabaco e HAS (Tabela 5).

**Tabela 5:** – Características após o pareamento em pacientes submetidos à cirurgia de revascularização do miocárdio

Características após o pareamento	Mulheres (n=1.947)		Homens (n=1.947)		Valor p
	n	%	n	%	
Idade (anos), média ± DP	66,70±10,03		66,76±9,53		0,895
Altura (m), média ± DP	1,57±0,07		1,70±0,07		<0,001
Peso (kg), média ± DP	66,76±12,30		78,27±12,99		<0,001
IMC, média ± DP	26,90±4,87		26,81±4,26		0,193
Uso de tabaco, n (%)	322	16,54	467	23,99	<0,001
HAS, n (%)	1.560	80,12	1.410	72,42	<0,001
DM, n (%)	662	34	659	33,85	0,919
Dislipidemia, n (%)	1.166	59,89	1.094	56,19	0,019
DPOC, n (%)	18	0,92	15	0,77	0,599
Classe CCS de angina, n (%)					
Classe 1	745	38,26	769	39,5	0,509
Classe 2	507	26,04	507	26,04	
Classe 3	295	15,15	307	15,77	
Classe 4	400	20,54	364	18,7	
Classe NYHA de dispneia, n (%)					
Classe I	972	49,92	998	51,26	0,493
Classe II	579	29,74	562	28,86	
Classe III	314	16,13	321	16,49	
Classe IV	82	4,21	66	3,39	
IM prévio (30 dias)	550	28,25	532	27,32	0,52
Insuficiência renal crônica (creatinina >2 mg/dL)	49	2,52	58	2,98	0,377
Doença arterial periférica	19	0,98	16	0,82	0,61
AVC prévio	65	3,34	66	3,39	0,929
CRM prévia	150	7,7	138	7,09	0,462
Uma reoperação	138	7,09	124	6,37	0,932
Duas ou três reoperações	12	0,62	13	0,67	
Classe de fração de ejeção					
Normal (>55%)	1.200	61,63	1.241	63,74	0,247
Leve (54-45%)	374	19,21	330	16,95	
Moderada (44-35%)	278	14,28	290	14,89	
Grave (<35%)	95	4,88	86	4,42	
Urgência ou emergência	81	4,16	74	3,8	0,566
Cirurgia associada	336	17,26	334	17,15	0,9323
Utilização de circulação extracorpórea	1.825	93,73	1.845	94,76	0,1686

AVC: acidente vascular cerebral; CCS: Canadian Cardiovascular Society – classificação de angina; CRM: cirurgia de revascularização do miocárdio; DM: diabetes melito; DPOC: doença pulmonar obstrutiva crônica; DP: desvio padrão; HAS: hipertensão arterial sistêmica; IM: infarto do miocárdio; IMC: índice de massa corporal; NYHA: New York Heart Association – classificação de dispneia.

As mulheres continuaram apresentando maior tempo de internação hospitalar, porém a taxa de mortalidade deixou de ser significativamente mais elevada (Tabela 6).

**Tabela 6:** – Características após o pareamento em pacientes submetidos à cirurgia de revascularização do miocárdio

Características	Mulheres (n=1.947)		Homens (n=1.947)		Valor p
Complicações após a cirurgia, n (%)	735	37,75	778	39,96	0,157
Embolia venosa, n (%)	21	1,08	20	1,03	0,875
Balão intra-aórtico, n (%)	56	2,88	41	2,11	0,123
Coagulopatia, n (%)	18	0,92	26	1,34	0,225
IM durante a internação, n (%)	80	4,11	64	3,29	0,174
Coma, n (%)	16	0,82	3	0,15	0,003
AVC, n (%)	105	5,39	85	4,37	0,137
Insuficiência cardiorrespiratória, n (%)	29	1,49	30	1,54	0,896
Duração da permanência hospitalar (dias), média ± DP	14,84±10,96		13,93±10,05		0,001
Dias após a cirurgia, média ± DP	10,84±9,53		10,42±9,04		0,014
Duração da permanência na UTI (horas), média ± DP	85,40±133,42		81,58±105,81		0,252
Complicações intraoperatórias, n (%)	222	11,4	193	9,91	0,132
Óbito, n (%)	93	4,78	78	4,01	0,241

AVC: acidente vascular cerebral; DP: desvio padrão; IM: infarto do miocárdio; UTI: unidade de terapia intensiva.

## Discussão

Os principais achados deste estudo evidenciaram diferenças entre homens e mulheres submetidos à CRM quanto às características pré-operatórias, complicações pós-operatórias e taxas de mortalidade. Após o controle da heterogeneidade das características pré-operatórias por meio do pareamento por escore de propensão, a duração da internação hospitalar manteve-se maior entre as mulheres, enquanto as taxas de mortalidade não apresentaram diferença significativa entre os grupos. No entanto, na identificação dos fatores prognósticos por meio da análise de regressão logística multivariada, o sexo feminino demonstrou forte associação com maior risco de mortalidade intra-hospitalar.

Comparar dois grupos com diferentes características basais ajuda a explicar a observação de resultados distintos. Neste estudo, observamos que as mulheres apresentaram maior mortalidade, mas também tinham maior prevalência de outros fatores prognósticos. Para isolar o efeito do sexo de possíveis fatores de confusão, utilizamos duas abordagens: o pareamento por escore de propensão e a regressão logística multivariada. Ao aplicar o pareamento por escore de propensão para equilibrar as características basais entre homens e mulheres, não encontramos diferença significativa na mortalidade intra-hospitalar. No entanto, ao isolar o sexo como variável de interesse na regressão logística multivariada, o sexo feminino se mostrou um fator prognóstico significativo para mortalidade operatória. Esse resultado controverso nos leva à reflexão sobre qual método deve ser priorizado na interpretação dos achados. Cepeda et al.^18^ realizaram uma comparação entre essas duas estratégias e demonstraram que o pareamento por escore de propensão apresenta maior poder empírico do que a regressão logística multivariada apenas quando há sete ou menos eventos por fator de confusão. No presente estudo, o número de eventos por fator de confusão foi significativamente superior a esse limiar, o que torna a regressão logística multivariada o método mais apropriado para sustentar nossa conclusão.

A associação entre o sexo feminino e a mortalidade operatória em cirurgias cardíacas, especialmente na CRM, é reconhecida há décadas.^19,20^ Modelos consagrados de estimativa de risco, como o modelo europeu EuroSCORE II e o *score* americano da *Society of Thoracic Surgeons* (STS), já consideram o sexo feminino como um fator de risco.^21,22^ No entanto, as opiniões sobre essa associação permanecem divididas.

Por exemplo, no modelo chinês de estimativa de risco (SinoSCORE), o sexo não é incluído no cálculo de risco.^23^ Uma análise com mais de 35.000 pacientes submetidos à CRM em Pequim^24^ mostrou que as mulheres apresentaram maiores taxas de mortalidade intra-hospitalar em comparação aos homens (1,62% vs 1,30%, p=0,0248). No entanto, após a aplicação da regressão logística multivariada, a idade avançada — e não o sexo feminino — foi identificada como fator prognóstico independente para mortalidade intra-hospitalar pós-CRM. Esses achados sugerem que fatores locais, genéticos e/ou sociais podem influenciar os desfechos, ressaltando a importância de se realizarem estudos semelhantes em diferentes contextos ao redor do mundo.

Outro estudo realizou uma análise secundária do ensaio GOPCABE, utilizando o conjunto de dados da população original para avaliar a influência do sexo feminino nos desfechos perioperatórios.^25^ A regressão logística multivariada indicou que o sexo feminino não parece ser um fator prognóstico para mortalidade em 30 dias após a CRM (OR: 0,703, IC 95%: 0,397-1,244, p=0,279). No entanto, essa análise apresenta uma limitação comum a todas as análises secundárias de ensaios clínicos randomizados: a maioria desses estudos recruta apenas uma pequena parcela dos pacientes atendidos na prática clínica cotidiana. Por isso, os achados podem não ser representativos do restante da população, limitando a generalização das comparações entre homens e mulheres.

A idade geralmente é considerada um fator de risco, em conjunto com o sexo. Em um estudo recente que analisou 30 anos de experiência com revascularização do miocárdio em um único país — a Dinamarca —, observou-se que as mulheres apresentaram maior mortalidade em 30 dias, 1 ano e 10 anos, em comparação aos homens.^13^ Um achado marcante foi que mulheres mais jovens apresentaram estimativas de mortalidade mais elevadas do que mulheres mais velhas. Esse resultado remete à influência de componentes biológicos, como a menopausa e a terapia de reposição hormonal, que podem estar associados à mortalidade cardiovascular e ter um impacto comparável ao da idade.^25^

No presente estudo, o IMC apresentou-se como um fator protetor, embora com limite superior do IC 95% de 0,99. Alguns estudos, no entanto, concluíram que pacientes obesos submetidos à CRM apresentam maior morbidade pós-operatória e pior sobrevida em longo prazo.^26^ O IMC elevado está fortemente associado a condições como DM, dislipidemia, HAS e fatores inflamatórios, como a leptina.^27^ Esses fatores estão relacionados à DAC, que há décadas se mantém como a principal causa de morte em adultos em todo o mundo.^1^ Entretanto, quando se observa apenas pacientes com DAC submetidos à CRM, estudos apontam que indivíduos com baixo peso são mais vulneráveis a complicações pós-operatórias que evoluem para óbito operatório.^28^ Além disso, ao incluir o IMC em modelos de regressão juntamente com outros fatores prognósticos, isolando sua influência, os resultados podem indicar que, quanto maior o IMC, menor o risco de mortalidade operatória.^23^ Por outro lado, uma terceira linha de evidência aponta uma perspectiva diferente: uma análise retrospectiva do banco de dados cirúrgico do *Royal Papworth Hospital* demonstrou que o IMC não se associa à mortalidade operatória, mas sim à redução da sobrevida tardia dos pacientes submetidos à CRM.^29^

Este estudo apresenta algumas limitações. A análise incluiu quase 10.000 pacientes cirúrgicos, mas os dados foram obtidos de um único hospital. Para alcançar esse número de pacientes, foram utilizados dados acumulados ao longo de 27 anos de prática cirúrgica. Testes laboratoriais e dados de exames complementares não foram incluídos, devido à ausência dessas informações no banco de dados. Trata-se de um estudo observacional, com foco exclusivo nos dados intra-hospitalares, o que o torna suscetível a fatores de confusão. Além disso, não houve acompanhamento após a alta hospitalar, de modo que os desfechos avaliados se restringem ao período intra-hospitalar. Os resultados também não permitem afirmar, de forma definitiva, que o IMC seja um fator prognóstico protetor para mortalidade operatória em cirurgia cardíaca. O IC 95% próximo de 1 sugere a possibilidade de erro estatístico tipo II, especialmente quando considerado em conjunto com as demais limitações mencionadas.

Apesar dessas limitações, os dados apresentados neste estudo são consistentes com outras evidências disponíveis na literatura. A análise de grandes bancos de dados continua sendo uma abordagem válida, pois permite comparações com a população geral e contribui com informações relevantes para este importante campo de estudo na cardiologia.^30,31^

## Conclusão

Mulheres apresentaram maior tempo de internação e maior mortalidade operatória. Embora a regressão logística multivariada tenha identificado o sexo feminino como fator prognóstico para mortalidade, após o pareamento por escore de propensão, essa diferença não se manteve significativa. Esses achados sugerem que a redução das disparidades pode contribuir para a melhora dos desfechos em mulheres submetidas à CRM.

## References

[1] Paez RP, Hossne NA, Santo JADE, Berwanger O, Santos RHN, Kalil RAK (2019). Coronary Artery Bypass Surgery in Brazil: Analysis of the National Reality Through the BYPASS Registry. Braz J Cardiovasc Surg.

[2] Brasil, Ministério da Saúde (2025). DATASUS.

[3] Evora PRB (2020). Cardiopulmonary Bypass in Myocardial Revascularization Surgery in the State of São Paulo. The REPLICCAR Study. Arq Bras Cardiol.

[4] Orlandi BMM, Mejia OAV, Borgomoni GB, Goncharov M, Rocha KN, Bassolli L (2020). REPLICCAR II Study: Data Quality Audit in the Paulista Cardiovascular Surgery Registry. PLoS One.

[5] Santos CA, Oliveira MA, Brandi AC, Botelho PH, Brandi JC, Santos MA (2014). Risk Factors for Mortality of Patients Undergoing Coronary Artery Bypass Graft Surgery. Rev Bras Cir Cardiovasc.

[6] Oliveira EL, Westphal GA, Mastroeni MF (2012). Características Clínico-Demográficas de Pacientes Submetidos a Cirurgia de Revascularização do Miocárdio e sua Relação com a Mortalidade. Braz J Cardiovasc Surg.

[7] Khalil KH, Sá MPBO, Vervoort D, Roever L, Pires MAA, Lima JMO (2021). Coronary Artery Bypass Graft Surgery in Brazil from 2008 to 2017. J Card Surg.

[8] Oliveira GMM, Brant LCC, Polanczyk CA, Malta DC, Biolo A, Nascimento BR (2022). Cardiovascular Statistics - Brazil 2021. Arq Bras Cardiol.

[9] Bechtel AJ, Huffmyer JL (2020). Gender Differences in Postoperative Outcomes after Cardiac Surgery. Anesthesiol Clin.

[10] The Society of Thoracic Surgeons (2023). Adult Cardiac Surgery Database Executive Summary 10 Years.

[11] Bukkapatnam RN, Yeo KK, Li Z, Amsterdam EA (2010). Operative Mortality in Women and Men Undergoing Coronary Artery Bypass Grafting (from the California Coronary Artery Bypass Grafting Outcomes Reporting Program). Am J Cardiol.

[12] Robinson NB, Naik A, Rahouma M, Morsi M, Wright D, Hameed I (2021). Sex Differences in Outcomes Following Coronary Artery Bypass Grafting: A Meta-Analysis. Interact Cardiovasc Thorac Surg.

[13] Adelborg K, Horváth-Puhó E, Schmidt M, Munch T, Pedersen L, Nielsen PH (2017). Thirty-Year Mortality after Coronary Artery Bypass Graft Surgery: A Danish Nationwide Population-Based Cohort Study. Circ Cardiovasc Qual Outcomes.

[14] Brooks MM, Jones RH, Bach RG, Chaitman BR, Kern MJ, Orszulak TA (2000). Predictors of Mortality and Mortality from Cardiac Causes in the Bypass Angioplasty Revascularization Investigation (BARI) Randomized Trial and Registry. For the BARI Investigators. Circulation.

[15] Vogel B, Acevedo M, Appelman Y, Merz CNB, Chieffo A, Figtree GA (2021). The Lancet Women and Cardiovascular Disease Commission: Reducing the Global Burden by 2030. Lancet.

[16] Toumpoulis IK, Anagnostopoulos CE, Balaram SK, Rokkas CK, Swistel DG, Ashton RC (2006). Assessment of Independent Predictors for Long-Term Mortality Between Women and Men after Coronary Artery Bypass Grafting: Are Women Different from Men?. J Thorac Cardiovasc Surg.

[17] Jaghoori A, Lamin V, Jacobczak R, Worthington M, Edwards J, Viana F (2020). Sex Differences in Vascular Reactivity of Coronary Artery Bypass Graft Conduits. Heart Vessels.

[18] Cepeda MS, Boston R, Farrar JT, Strom BL (2003). Comparison of Logistic Regression versus Propensity Score When the Number of Events is Low and There are Multiple Confounders. Am J Epidemiol.

[19] Abramov D, Tamariz MG, Sever JY, Christakis GT, Bhatnagar G, Heenan AL (2000). The Influence of Gender on the Outcome of Coronary Artery Bypass Surgery. Ann Thorac Surg.

[20] King KB, Clark PC, Hicks GL (1992). Patterns of Referral and Recovery in Women and Men Undergoing Coronary Artery Bypass Grafting. Am J Cardiol.

[21] Nashef SA, Roques F, Sharples LD, Nilsson J, Smith C, Goldstone AR (2012). EuroSCORE II. Eur J Cardiothorac Surg.

[22] Kouchoukos NT, Ebert PA, Grover FL, Lindesmith GG (1988). Report of the Ad Hoc Committee on Risk Factors for Coronary Artery Bypass Surgery. Ann Thorac Surg.

[23] Zheng Z, Zhang L, Chinese Cardiovascular Surgery Registry (2010). Chinese Risk Stratification Scoring System for Coronary Artery Bypass Grafting. Zhonghua Xin Xue Guan Bing Za Zhi.

[24] Wang J, Yu W, Zhao D, Liu N, Yu Y (2017). In-Hospital and Long-Term Mortality in 35,173 Chinese Patients Undergoing Coronary Artery Bypass Grafting in Beijing: Impact of Sex, Age, Myocardial Infarction, and Cardiopulmonary Bypass. J Cardiothorac Vasc Anesth.

[25] Schipper I, Louwers YV (2020). Premature and Early Menopause in Relation to Cardiovascular Disease. Semin Reprod Med.

[26] Habib RH, Zacharias A, Schwann TA, Riordan CJ, Durham SJ, Shah A (2005). Effects of Obesity and Small Body Size on Operative and Long-Term Outcomes of Coronary Artery Bypass Surgery: A Propensity-Matched Analysis. Ann Thorac Surg.

[27] Mokdad AH, Ford ES, Bowman BA, Dietz WH, Vinicor F, Bales VS (2003). Prevalence of Obesity, Diabetes, and Obesity-Related Health Risk Factors, 2001. JAMA.

[28] Protopapas AD (2016). Does Body Mass Index Affect Mortality in Coronary Surgery?. Open Cardiovasc Med J.

[29] Benedetto U, Danese C, Codispoti M (2014). Obesity Paradox in Coronary Artery Bypass Grafting: Myth or Reality?. J Thorac Cardiovasc Surg.

[30] Lotufo PA, Malta DC, Szwarcwald CL, Stopa SR, Vieira ML, Bensenor IM (2015). Prevalência de Angina do Peito pelo Questionário de Rose na População Brasileira: Análise da Pesquisa Nacional de Saúde, 2013. Rev Bras Epidemiol.

[31] Lacava L, Freitas FL, Borgomoni GB, Silva PGMBE, Nakazone MA, Campagnucci VP (2024). More Hospital Complications in Women after Cabg Even for Reduced Surgical Times: Call to Action for Equity in Quality Improvement. Arq Bras Cardiol.

