# Supplementary material for: O Sexo Feminino está Associado à Mortalidade na Cirurgia de Revascularização do Miocárdio?
Source: Arq Bras Cardiol. 2025 May 8;122(5):e20240664. [Article in Portuguese] doi: 10.36660/abc.20240664 (PMC12129472; doi:10.36660/abc.20240664)
Supplement: Supplementary file 1 [file 0066-782x-abc-122-5-e20240664-suppl01.pdf]

SUPLEMENTAR

Tabela 1. Diferenças das médias antes e após o escore de propensão

Variable	Std. Mean Diff. Before PSM	Std. Mean Diff. After PSM
distance	0.5118	0.0086
Idade	0.4492	-0.0058
IMC	-0.1508	0.0195
Cirassociada	0.1541	0.0027
DPOC	-0.0489	0.0161
Urgencia	0.0688	0.0180
IAM30d	-0.0863	0.0205
CCS	0.1776	0.0375
NYHA	0.1695	0.0299
InsVasPeripre	0.0001	0.0157
revascprevia	-0.0848	0.0231
InsRenal	-0.0608	-0.0295
FEVE	-0.0260	0.0268

Tabela 2. Regressão univariada em pacientes submetidos à CRM

Variável	OR	IC 95% Inferior	IC 95% Superior	Valor de P
**Idade (anos)**	1,07	1,06	1,08	<0,001
**Sexo (feminino)**	2,01	1,56	2,58	<0,001
**IMC**	0,94	0,92	0,96	<0,001
**Cirurgia associada**	4,77	3,72	6,09	<0,001
**Diabetes mellitus**	1,11	0,86	1,42	0,403
**Doença pulmonar crônica**	2,59	1,21	4,87	0,007
**Urgência ou emergência**	4,89	3,31	7,02	<0,001
**IM recente (30 dias)**	1,64	1,29	2,08	<0,001
**CCS**				
2	1,04	0,75	1,43	<0,001
3	1,51	1,06	2,12	<0,001
4	1,99	1,46	2,71	<0,001
**NYHA**				
II	1,5	1,11	2,01	<0,001
III	2,55	1,85	3,49	<0,001
IV	9,55	6,44	13,94	<0,001
**Doença arterial periférica**	2,26	0,88	4,79	0,055
**CRM prévia**	3,2	2,4	4,21	<0,001
**Fração de ejeção**				
45-54%	1,44	1,01	2,02	<0,001
35-44%	2,51	1,83	3,42	<0,001

<35%	8,5	6,12	11,74	<0,001
**Insuficiência renal (Creatinina >2 mg/dL)**	3,28	2,12	4,86	<0,001

Tabela 3. Fatores de predição de risco selecionados para regressão logística multivariada

Variáveis	EuroSCORE II	SinoSCORE	InsCor	Dados HCor	Regressão Multivariada
Idade	+	+	+	+	+
Sexo	+	-	+	+	+
IMC	-	+	-	+	+
Insuficiência renal	+	+	+	+	+
Arteriopatia extracardíaca	+	+	-	+	+
Mobilidade limitada	+	-	-	-	-
Cirurgia cardíaca prévia	+	-	+	CRM prévia	+
Doença pulmonar crônica	+	+	-	+	+
Endocardite ativa	+	-	-	-	-
Estado crítico	+	+	+	-	-
Diabetes mellitus	Terapia com insulina	-	-	+	-
NYHA	+	3 ou 4	-	+	+
CCS	4	-	-	+	+
Fração de ejeção	+	+	<30%	+	+
IM prévio	90 dias	-	90 dias	30 dias	+
Hipertensão pulmonar	+	-	-	-	-
Urgência ou emergência	+	+	-	+	+
Cirurgia associada	+	+	+	+	+
Cirurgia de aorta torácica	+	-	-	-	-
Cirurgia de válvula aórtica	-	-	+	-	-
Cirurgia de válvula tricúspide	-	-	+	-	-
Fibrilação/Flutter atrial	-	+	-	-	-

IMC: Índice de massa corpórea; IM: Infarto do miocárdio; CCS: The Canadian Cardiovascular Society angina classification; NYHA: New York Heart Association dyspnea classification; CRM: Cirurgia de revascularização do miocárdio;
