# Supplementary material for: O Sexo Feminino está Associado à Mortalidade na Cirurgia de Revascularização do Miocárdio?
Source: Arq Bras Cardiol. 2025 May 8;122(5):e20240664. [Article in Portuguese] doi: 10.36660/abc.20240664 (PMC12129472; doi:10.36660/abc.20240664)
Supplement: Translation

# Translation: Is Female Gender Associated with Mortality in Coronary Artery Bypass Grafting?

**DOI:** 10.36660/abc.20240664i

**Authors:** Maxim Goncharov, Erlon Oliveira de Abreu Silva, Pedro Gabriel Melo de Barros e Silva, Fabiane Letícia de Freitas, Adriana Costa Moreira, Lucas Tramujas, Alexandre Biasi Cavalcanti, Ieda Maria Liguori, Fabio Biscegli Jatene, Ieda Biscegli Jatene, Claudia Maria Rodrigues Alves

**Affiliations:** 1 Brazil Hospital do Coração, São Paulo, SP – Brazil; 2 Brazil Instituto do Coração do Hospital das Clínicas da Faculdade de Medicina da Universidade de São Paulo, São Paulo, SP – Brazil

**Keywords:** Hospital Mortality, Cardiovascular Surgical Procedures, Women

## Abstract

**Background:**

Women undergoing coronary artery bypass grafting (CABG) tend to have worse in-hospital outcomes, but it is unclear whether these differences are due to gender or to clinical factors.

**Objective:**

To compare in-hospital outcomes between women and men undergoing CABG.

**Methods:**

This was a single-center, retrospective observational study analyzing data from 9,845 patients who underwent CABG between 1995 and 2022, of whom 1,947 (19.8%) were women. To evaluate female gender as a prognostic factor for in-hospital mortality, we used descriptive statistics, univariate and multivariate logistic regression, and propensity score matching. The significance level was set at 5%.

**Results:**

Women were older (66.7 vs 62.19 years, p<0.001), had lower body mass index (26.91 vs 27.64, p<0.001), and had a higher prevalence of diabetes mellitus (34.0% vs 31.6%, p=0.045). They also had longer hospital stays (14.84 vs 13.13 days, p<0.001) and higher operative mortality (4.8% vs 2.4%, p<0.001). In logistic regression, female gender was associated with higher mortality (OR=1.51; 95% CI: 1.15-1.99; p=0.003). After matching, there was no significant difference in mortality (OR=1.20; 95% CI: 0.88-1.64; p=0.241), but length of hospital stay remained longer in women.

**Conclusion:**

When clinical factors were matched between men and women, the mortality difference disappeared. This suggests that targeted interventions to reduce disparities may help improve mortality outcomes in women undergoing CABG.

## Introduction

Cardiovascular disease is the leading cause of morbidity and mortality worldwide, with coronary artery disease (CAD) being the most common form.^1^ In developing countries, interpretation of data from national registries is challenging due to population heterogeneity and significant differences between public and private hospitals.^2-4^

Data on this issue in the Brazilian population are still scarce. Currently, 58% of revascularization procedures are performed by angioplasty, and the ratio between angioplasty and coronary artery bypass grafting (CABG) increased significantly from 2008 to 2019. This shift reflects a new patient profile, with characteristics that are less favorable for surgery, which may impact CABG outcomes.^5-8^

Circulatory system diseases caused more than 170,000 deaths in women in 2019.^1^ Gender has been shown to be an important factor in the treatment of ischemic heart disease. Overall, women tend to develop the disease later in life because of hormonal protection, which contributes to higher mortality rates in CABG.^9-13^ However, some studies have not found this difference.^14 1^4⁴ This discrepancy may be explained by the exclusion of gender-specific factors — such as early menopause, microvascular dysfunction, and differences in arterial diameter — in large registries and randomized trials.^15-17^

Because of the high incidence of CAD, especially in women, more national data are needed to fill the knowledge gap regarding these differences. Therefore, the aim of this study was to compare demographic, clinical, and surgical characteristics and outcomes between men and women as well as to assess the impact of female gender as an independent prognostic factor for mortality in CABG.

## Methods

### Study design

This was a retrospective observational study that analyzed a cohort of patients who underwent CABG at a private cardiac center (Central Illustration).

Since 1995, data from all surgical procedures performed in patients over 18 years of age who underwent CABG — whether elective or not, performed alone or combined with valve surgery, ventricular aneurysm repair, or ascending aorta surgery — have been collected daily by health care professionals. Data were stored in an electronic platform integrated with the Tasy^®^ hospital information system (Philips, Blumenau, SC, Brazil). They were stored on the hospital’s server, with access restricted to authorized staff from the institution.

To ensure data accuracy and quality, an indirect audit was performed by evaluating the consistency, completeness, and accuracy of information. In addition, the incidence of perioperative characteristics was analyzed year by year to confirm the reliability of variables. As a result, 136 patients with incomplete data were excluded from the final analysis (Figure 1).

**Figure 1:** – Flowchart of patient selection for inclusion in the study. CABG: coronary artery bypass grafting.

### Statistical analysis

All analyses were performed using R software, version 4.0.1.

Categorical variables were reported as absolute numbers and percentages, while continuous variables were presented as means and standard deviations (SD). The normality of continuous variables was assessed using the Anderson-Darling test, and the homogeneity of variances was tested using Levene’s test.

For group comparisons, all continuous variables had a normal distribution and were analyzed using the unpaired Student’s t-test to compare means. Categorical variables were compared using Fisher’s exact test or the chi-square test as appropriate.

An additional sensitivity analysis was performed using propensity score matching to minimize potential bias between the groups. Variables included in the matching process were age, body mass index (BMI), combined surgery, chronic obstructive pulmonary disease (COPD), urgent or emergency status, myocardial infarction (MI) within the past 30 days, angina class according to the Canadian Cardiovascular Society (CCS), dyspnea class according to the New York Heart Association (NYHA), peripheral artery disease, prior CABG, chronic kidney disease, and ejection fraction class.

Univariate and multivariate logistic regressions were then performed to identify independent risk factors for in-hospital mortality, considering female gender as one of the risk variables, along with the other factors. The significance level was set at 5%.

## Results

Significant differences were observed in baseline characteristics between men and women (Table 1). Women were older, had lower BMI, and showed a higher prevalence of SAH and diabetes mellitus (DM). They also presented with more advanced functional classes of dyspnea and angina. However, compared to men, they had lower rates of recent MI, chronic kidney disease, and prior cardiac surgeries.

**Table 1:** – Preoperative characteristics of patients undergoing coronary artery bypass grafting

Characteristics	Women (n=1,947)		Men (n=7,898)		p-value
Age (years), mean ± SD	66.7±10.03		62.19±10.15		<0,001
Height (m), mean ± SD	1.57±0.07		1.71±0.07		<0,001
Weight (kg), mean ± SD	66.76±12.3		80.85±13.81		<0,001
BMI, mean ± SD	26.91±4.87		27.64±4.28		<0,001
Tobacco use, n (%)	322	(16.5)	2,133	(27)	<0.001
SAH, n (%)	1,560	(80.1)	5,662	(71.7)	<0.001
DM, n (%)	662	(34.0)	2,499	(31.6)	0.045
Dyslipidemia, n (%)	1,166	(59.9)	4,603	(58.3)	0.200
COPD, n (%)	18	(0.9)	110	(1.4)	0.130
CCS angina class, n (%)					
Class 1	745	(38.3)	3,635	(46.0)	<0.001
Class 2	507	(26.0)	1,993	(25.2)	
Class 3	295	(15.2	1,099	(13.9)	
Class 4	400	(20.5)	1,171	(14.8)	
NYHA dyspnea class, n (%)					
Class I	972	(49.9)	4,565	(57.8)	<0.001
Class II	579	(29.7)	2,124	(26.9)	
Class III	314	(16.1)	1,028	(13.0)	
Class IV	82	(4.2)	181	(2.3)	
Prior MI (within 30 days), n (%)	550	(28.2)	2.538	(32.1)	0.001
Chronic kidney disease (creatinine >2 mg/dL), n (%)	49	(2.5)	274	(3.5)	0.040
Peripheral artery disease, n (%)	19	(1.0)	77	(1.0)	0.900
Prior stroke, n (%)	65	(3.3)	211	(2.7)	0.100
Prior CABG, n (%)	150	(7.7)	787	(10.0)	0.002
One reoperation, n (%)	138	(7.1)	710	(9.0)	0.020
Two or three reoperations, n (%)	12	(0.6)	76	(1.0)	
Ejection fraction, n (%)					
Normal (>55%)	1.200	(61,6)	4.778	(60,5)	0,550
Mild (54-45%)	374	(19,2)	1.506	(19,1)	
Moderate (44-35%)	278	(14,3)	1.231	(15,6)	
Severe (<35%)	95	(4,9)	382	(4,8)	

BMI: body mass index; CABG: coronary artery bypass grafting; CCS: Canadian Cardiovascular Society; COPD: chronic obstructive pulmonary disease; DM: diabetes mellitus; MI: myocardial infarction; NYHA: New York Heart Association; SAH: systemic arterial hypertension.

Intraoperative characteristics (Table 2) showed a higher frequency of nonelective surgeries and combined procedures in women, especially valve surgeries. Women also had a lower use of cardiopulmonary bypass. Intraoperative complications such as arrhythmias, bleeding, and low cardiac output syndrome were more common in the female group.

**Table 2:** – Intraoperative characteristics of patients undergoing coronary artery bypass grafting

Intraoperative characteristics	Women (n=1,947)		Men (n=7,898)		p-value
	n	%	n	%	
Urgent or emergency status	81	4.2	220	2.8	0.002
Combined surgery	337	17.3	908	11.4	<0.001
Valve surgery	209	10.7	534	6.8	<0.001
Aortic surgery	2	0.1	1	0.01	0.100
Aortic and valve surgery	0	0	2	0	1
Other surgeries	126	6.5	371	4.7	<0.001
Use of cardiopulmonary bypass	1,825	93.7	7,504	95	0.020
Intraoperative complications	222	11.4	764	9.7	0.020

Postoperative complications (Table 3) were similar between men and women in terms of ICU length of stay. However, women had a longer length of hospital stay. Although the overall rate of postoperative complications was comparable between groups, in-hospital mortality was significantly higher in women.

**Table 3:** – Postoperative complications in patients undergoing coronary artery bypass grafting

Postoperative complications	Women (n=1,947)		Men (n=7,898)		p-value
Complications, n (%)	735	(37.8)	2,804	(35.5)	0.060
Venous embolism, n (%)	21	(1.1)	77	(1.0)	0.670
Intra-aortic balloon pump, n (%)	56	(2.9)	154	(1.9)	0.010
Coagulopathy, n (%)	18	(0.9)	74	(0.9)	0.900
MI during hospitalization, n (%)	80	(4.1)	268	(3.4)	0.130
Coma, n (%)	16	(0.8)	10	(0.1)	<0.001
Stroke, n (%)	105	(5.4)	230	(2.9)	<0.001
Cardiorespiratory failure, n (%)	29	(1.5)	74	(0.9)	0.030
Length of hospital stay (days), mean ± SD	14.84±10.96		13.13±8.5		<0.001
Days after surgery, mean ± SD	10.84±9.53		9.73±7.19		<0.001
Length of ICU stay (hours), mean ± SD	85.4±133.42		74.85±84.61		0.400
Death, n (%)	93	(4.8)	192	(2.4)	<0.001

ICU: intensive care unit; MI: myocardial infarction; SD: standard deviation.

Univariate logistic regression showed that variables such as gender, age, BMI, combined surgery, urgent or emergency status, prior MI, angina class, dyspnea class, prior CABG, COPD, chronic kidney disease, and ejection fraction class were statistically significant (Supplementary Table 2). Peripheral artery disease was not statistically significant but was still included in the multivariate analysis along with the other significant variables. Based on these findings and the main risk models (EuroSCORE II, SinoSCORE, and InsCor) (Supplementary Table 3), 13 variables were selected for the multivariate logistic regression (Table 4).

**Table 4:** – Multivariate regression in patients undergoing coronary artery bypass grafting

Variable	OR	95% CI (lower)	95% CI (upper)	p-value
Age (years)	1.05	1.04	1.07	<0.001
Gender (female)	1.52	1.15	1.99	0.003
BMI	0.96	0.94	0.99	0.007
Combined surgery	3.02	2.28	3.98	<0.001
COPD	2.47	1.11	4.86	0.015
Urgent or emergency status	3.7	2.34	5.69	<0.001
Prior MI (within 30 days)	1.15	0.87	1.51	0.322
CCS angina class, n (%)				
Class 2	1.11	0.78	1.56	0.568
Class3	1.27	0.87	1.84	0.204
Class 4	1.6	1.14	2.23	0.006
NYHA dyspnea class, n (%)				
Class II	1.12	0.81	1.54	0.494
Class III	1.31	0.91	1.87	0.136
Class IV	2.48	1.55	3.9	<0.001
Peripheral artery disease	1.92	0.71	4.33	0.153
Prior CABG	2.14	1.56	2.89	<0.001
Ejection fraction class				
45-54%	1.24	0.86	1.76	0.24
35-44%	1.74	1.24	2.43	0.001
<35%	3.4	2.26	5.08	<0.001
Chronic kidney disease (creatinine >2 mg/dL)	1.91	1.17	2.99	0.007

BMI: body mass index; CABG: coronary artery bypass grafting; CCS: Canadian Cardiovascular Society; COPD: chronic obstructive pulmonary disease; MI: myocardial infarction; NYHA: New York Heart Association; OR: odds ratio.

After multivariate logistic regression, variables such as age, combined surgery, urgent or emergency status, recent MI, angina class, dyspnea class, prior CABG, COPD, chronic kidney disease, and ejection fraction class were associated with a higher risk of mortality. BMI was found to be a protective factor, while female gender was also significantly associated with increased mortality.

After propensity score matching, with 1,947 patients in each group, most preoperative and intraoperative characteristics were similar, except for tobacco use and SAH (Table 5).

**Table 5:** – Characteristics after matching in patients undergoing coronary artery bypass grafting (CABG)

Characteristics after matching	Women (n=1,947)		Men (n=1,947)		p-value
	n	%	n	%	
Age (years), mean ± SD	66.70±10.03		66.76±9.53		0.895
Height (m), mean ± SD	1.57±0.07		1.70±0.07		<0.001
Weight (kg), mean ± SD	66.76±12.30		78.27±12.99		<0.001
BMI, mean ± SD	26.90±4.87		26.81±4.26		0.193
Tobacco use, n (%)	322	16.54	467	23.99	<0,001
SAH, n (%)	1,560	80.12	1,410	72.42	<0,001
DM, n (%)	662	34	659	33.85	0,919
Dyslipidemia, n (%)	1,166	59.89	1,094	56.19	0,019
COPD, n (%)	18	0.92	15	0.77	0,599
CCS angina class, n (%)					
Class 1	745	38.26	769	39.5	0,509
Class 2	507	26.04	507	26.04	
Class 3	295	15.15	307	15.77	
Class 4	400	20.54	364	18.7	
NYHA dyspnea class, n (%)					
Class I	972	49.92	998	51.26	0,493
Class II	579	29.74	562	28.86	
Class III	314	16.13	321	16.49	
Class IV	82	4.21	66	3.39	
Prior MI (within 30 days)	550	28.25	532	27.32	0,52
Chronic kidney disease (creatinine >2 mg/dL)	49	2.52	58	2.98	0.377
Peripheral artery disease	19	0.98	16	0.82	0.61
Prior stroke	65	3.34	66	3.39	0.929
Prior CABG	150	7.7	138	7.09	0.462
One reoperation	138	7.09	124	6.37	0.932
Two or three reoperations	12	0.62	13	0.67	
Ejection fraction class					
Normal (>55%)	1,200	61.63	1,241	63.74	0.247
Mild (54–45%)	374	19.21	330	16.95	
Moderate (44–35%)	278	14.28	290	14.89	
Severe (<35%)	95	4.88	86	4.42	
Urgent or emergency status	81	4.16	74	3.8	0.566
Combined surgery	336	17.26	334	17.15	0.9323
Use of cardiopulmonary bypass	1,825	93.73	1,845	94.76	0.1686

BMI: body mass index; CABG: coronary artery bypass grafting; CCS: Canadian Cardiovascular Society; COPD: chronic obstructive pulmonary disease; DM: diabetes mellitus; MI: myocardial infarction; NYHA: New York Heart Association; SD: standard deviation.

Women continued to have longer hospital stays, but the mortality rate was no longer significantly higher (Table 6).

**Table 6:** – Post-matching outcomes in patients undergoing coronary artery bypass grafting

Characteristics	Women (n=1,947)		Men (n=1,947)		p-value
Complications after surgery, n (%)	735	37.75	778	39.96	0.157
Venous embolism, n (%)	21	1.08	20	1.03	0.875
Intra-aortic balloon pump, n (%)	56	2.88	41	2.11	0.123
Coagulopathy, n (%)	18	0.92	26	1.34	0.225
MI during hospitalization, n (%)	80	4.11	64	3.29	0.174
Coma, n (%)	16	0.82	3	0.15	0.003
Stroke, n (%)	105	5.39	85	4.37	0.137
Cardiorespiratory failure, n (%)	29	1.49	30	1.54	0.896
Length of hospital stay (days), mean ± SD	14.84±10.96		13.93±10.05		0.001
Days after surgery, mean ± SD	10.84±9.53		10.42±9.04		0.014
Length of ICU stay (hours), mean ± SD	85.40±133.42		81.58±105.81		0.252
Intraoperative complications, n (%)	222	11.4	193	9.91	0.132
Death, n (%)	93	4.78	78	4.01	0.241

ICU: intensive care unit; MI: myocardial infarction; SD: standard deviation.

## Discussion

The main findings of this study showed differences between men and women who underwent CABG in terms of preoperative characteristics, postoperative complications, and mortality rates. After adjusting for differences in baseline characteristics using propensity score matching, women still had longer hospital stays, but mortality rates were no longer significantly different between the groups. However, in the multivariable logistic regression analysis used to identify prognostic factors, female gender remained strongly associated with a higher risk of in-hospital mortality.

Comparing two groups with different baseline characteristics helps explain why their outcomes may differ. In this study, we found that women had higher mortality, but they also had a higher prevalence of other risk factors. To isolate the effect of gender from potential confounding factors, we used two approaches: propensity score matching and multivariable logistic regression. When we applied propensity score matching to balance the baseline characteristics between men and women, there was no significant difference in in-hospital mortality. However, when gender was analyzed as an independent variable in the multivariable logistic regression model, the female gender emerged as a significant predictor of operative mortality. This conflicting result raises the question of which method should be prioritized when interpreting the findings. Cepeda et al.^18^ compared these two strategies and showed that propensity score matching only has greater empirical power than multivariable logistic regression when there are seven or fewer events per confounding variable. In our study, the number of events per confounder was well above that threshold, making multivariable logistic regression the more appropriate method to support our conclusion.

The link between female gender and operative mortality in cardiac surgery — particularly in CABG — has been acknowledged for decades.^19,20^ Established risk prediction models, such as the European EuroSCORE II and the U.S. Society of Thoracic Surgeons (STS) score, already include female gender as a risk factor.^21,22^ However, opinions on this association remain divided.

For example, in the Chinese risk prediction model (SinoSCORE), gender is not included as a risk factor.^23^ An analysis of more than 35,000 patients who underwent CABG in Beijing^24^ showed that women had higher in-hospital mortality rates compared to men (1.62% vs 1.30%, p=0.0248). However, after applying multivariable logistic regression, increased age — not female gender — was found to be an independent predictor of in-hospital mortality after CABG. Such findings suggest that local, genetic, and/or social factors may influence outcomes, underscoring the need for similar studies across diverse populations worldwide.

Another study conducted a secondary analysis of the GOPCABE trial using data from the original study population to assess the impact of female gender on perioperative outcomes.^25^ The multivariable logistic regression showed that female gender did not appear to be a predictor of 30-day mortality after CABG (OR: 0.703, 95% CI: 0.397-1.244, p=0.279). However, this analysis shares a common limitation with all secondary analyses of randomized clinical trials: most trials enroll only a small portion of the patients typically treated in everyday clinical practice. As a result, the findings may not represent the broader population, limiting the generalizability of comparisons between men and women.

Age is usually considered a risk factor alongside gender. In a recent study analyzing 30 years of experience with CABG in a single country — Denmark — women showed higher mortality rates at 30 days, 1 year, and 10 years compared to men.^13^ A striking finding was that younger women had higher estimated mortality rates than older women. This points to the possible influence of biological factors, such as menopause and hormone replacement therapy, which may be linked to cardiovascular mortality and have an impact comparable to that of age.^25^

In the present study, BMI appeared as a protective factor, although the upper limit of the 95% CI was 0.99. However, some studies have found that obese patients undergoing CABG experience higher postoperative morbidity and worse long-term survival.^26^ Increased BMI is strongly associated with conditions such as DM, dyslipidemia, SAH, and inflammatory markers such as leptin.^27^ These factors are linked to CAD, which has remained the leading cause of death among adults worldwide for decades.^1^ When focusing specifically on patients with CAD undergoing CABG, some studies have shown that underweight individuals are more vulnerable to postoperative complications that can lead to operative death.^28^ Moreover, when BMI is included in regression models along with other prognostic factors — allowing its effect to be isolated — the results may suggest that higher BMI is associated with a lower risk of operative mortality.^23^ On the other hand, a third line of evidence offers a unique perspective: a retrospective analysis of the surgical database from the Royal Papworth Hospital found no association between BMI and operative mortality, but did show reduced long-term survival among patients with higher BMI undergoing CABG.^29^

This study has several limitations. Although it included nearly 10,000 surgical patients, data came from just one hospital. To reach that number, data were gathered over 27 years of surgical practice. Laboratory tests and imaging data were not included because they were not available in the database. Since this was an observational study based only on in-hospital data, the results may have been affected by other confounders not accounted for. In addition, there was no follow-up after patients were discharged from the hospital, so all outcomes are limited to the in-hospital period. The results also do not allow us to say for certain that BMI is a protective factor for operative mortality in cardiac surgery. The 95% CI being close to 1 suggests there may be a type II statistical error, especially when considered along with the other limitations already mentioned.

Despite these limitations, data in this study align with other evidence in literature. Analyzing large databases is still a valid approach because it allows for comparisons with the general population and offers valuable insights into this important area of cardiology research.^30,31^

## Conclusion

Women had longer hospital stays and higher operative mortality. Although multivariable logistic regression identified female gender as a prognostic factor for mortality, this difference was no longer significant after propensity score matching. These findings suggest that reducing disparities may help improve outcomes in women undergoing CABG.
